# Carbon uptake by mature Amazon forests has mitigated Amazon nations’ carbon emissions

**DOI:** 10.1186/s13021-016-0069-2

**Published:** 2017-02-15

**Authors:** Oliver L. Phillips, Roel J. W. Brienen, the RAINFOR collaboration

**Affiliations:** School of Geography, University of Leeds, Leeds, LS2 9JT UK

**Keywords:** Amazonia, Carbon balance, Carbon sink, Sequestration, Land use change, Climate change, Tropical forests, Ecosystem service

## Abstract

**Background:**

Several independent lines of evidence suggest that Amazon forests have provided a significant carbon sink service, and also that the Amazon carbon sink in intact, mature forests may now be threatened as a result of different processes. There has however been no work done to quantify non-land-use-change forest carbon fluxes on a national basis within Amazonia, or to place these national fluxes and their possible changes in the context of the major anthropogenic carbon fluxes in the region. Here we present a first attempt to interpret results from ground-based monitoring of mature forest carbon fluxes in a biogeographically, politically, and temporally differentiated way. Specifically, using results from a large long-term network of forest plots, we estimate the Amazon biomass carbon balance over the last three decades for the different regions and nine nations of Amazonia, and evaluate the magnitude and trajectory of these differentiated balances in relation to major national anthropogenic carbon emissions.

**Results:**

The sink of carbon into mature forests has been remarkably geographically ubiquitous across Amazonia, being substantial and persistent in each of the five biogeographic regions within Amazonia. Between 1980 and 2010, it has more than mitigated the fossil fuel emissions of every single national economy, except that of Venezuela. For most nations (Bolivia, Colombia, Ecuador, French Guiana, Guyana, Peru, Suriname) the sink has probably additionally mitigated all anthropogenic carbon emissions due to Amazon deforestation and other land use change. While the sink has weakened in some regions since 2000, our analysis suggests that Amazon nations which are able to conserve large areas of natural and semi-natural landscape still contribute globally-significant carbon sequestration.

**Conclusions:**

Mature forests across all of Amazonia have contributed significantly to mitigating climate change for decades. Yet Amazon nations have not directly benefited from providing this global scale ecosystem service. We suggest that better monitoring and reporting of the carbon fluxes within mature forests, and understanding the drivers of changes in their balance, must become national, as well as international, priorities.

**Electronic supplementary material:**

The online version of this article (doi:10.1186/s13021-016-0069-2) contains supplementary material, which is available to authorized users.

## Background

Biospheric processes of carbon exchange exert significant control on the evolution of the atmospheric carbon dioxide burden, and hence on the rate of global climate change itself. Over recent decades on average less than half of anthropogenic carbon dioxide emissions have accumulated in the atmosphere, with the balance apportioned to large sinks of the order of ca. 2.5 Pg C yr^−1^ each in the oceans and on land [e.g., 7, 13]. Nevertheless, the terrestrial sink and the terrestrial fluxes, apart from those due to fossil fuel emissions, remain poorly constrained and are often computed simply as the residual of the better quantified fluxes into the ocean and those due to direct anthropogenic processes [e.g., 7]. The terrestrial sink also exhibits substantial inter-annual variation, which is largely driven by variations in temperature and moisture particularly in the tropics [e.g., 56, 57]. Both the large long-term terrestrial sink and its strong inter-annual variation indicate potentially critical roles for the planet’s most productive terrestrial ecosystems to modify and respond to anthropogenic climate change.

As the world’s largest tropical forest by extent the Amazon is a leading candidate for influencing the long-term terrestrial carbon balance and fluxes, their inter-annual fluctuations, and any trend in the terrestrial sink. Its remoteness challenges attempts to map and monitor its carbon function but several lines of measurement evidence illustrate the significance and climate sensitivity of its carbon fluxes. For example, eddy covariance measurements of canopy gas exchange suggest that the landscape-scale carbon balance of natural Amazon forests is seldom in balance on sub-annual timescales [e.g., 49], while aircraft measurements of atmospheric carbon dioxide concentrations and inverse modelling of the trajectory of air parcels reveal strong inter-annual differences and drought sensitivity at the basin-scale [24]. Satellite-based assessment of deforestation and fire confirm large but spatially and temporally very variable emissions from the loss of biomass [e.g., 5, 30, 53].

Permanent plots in which the lives of individual trees are tracked are a key technology for investigating the biomass fluxes and net balance of forests worldwide [e.g., 40]. On a per-unit-area basis, the net fluxes within mature forests are expected to be much smaller than these from deforestation, degradation, and regrowth processes, but such small changes in mature forests may nevertheless scale to large values when integrating over bigger regions. While efforts to track the behaviour of Amazon forests on the ground are sparser than in most temperate regions, the total on-the-ground monitoring effort has nevertheless increased several-fold since the early 1980’s, to encompass more than 300 plots by the 2000’s using standardized protocols. By the late 1990’s this long-term network was already suggesting that mature Amazon forests were not in balance [43]. The expanding measurement base has continued to support the inference of a large, long-term carbon sink into forest biomass, also showing that while the sink results from productivity exceeding mortality, both the rate of growth and the rate of death have tended to increase [e.g., 35], and that the sink extends beyond Amazonia to other tropical forests [e.g., 36]. Most recently, Amazon tree growth rates have stalled, but tree mortality has continued to accelerate, so that the net balance of the two—the biomass carbon sink—has declined [10]. The reasons for this continued increase in mortality remain uncertain. It has been proposed that faster growth may lead to faster tree death [e.g., 11, 42], while evidence also suggests that recent intense droughts in parts of Amazonia are directly responsible for killing enough trees to shut down the biomass sink for periods of a year or more [e.g., 51], via mechanisms such as carbon starvation or hydraulic failure [15, 50]. The ground data from the Amazon RAINFOR network are also consistent with atmospheric GHG profiles [24] in showing both the sensitivity of the carbon balance of intact Amazon forests to drought in 2005 and 2010, and the continued net sink of hundreds of millions of tons in non-drought years [20, 41].

In sum, observations indicate that the remaining old-growth forests in Amazonia have contributed a large net biomass sink from the atmosphere to the land, albeit one that appears to be in decline as a result of different processes. There has however remarkably been no effort to quantify such net fluxes on a regional or national basis within Amazonia, or to place them and their possible changes directly in the context of major anthropogenic carbon fluxes in the region. Addressing this major gap is important for at least three reasons. First, historically, if Amazonia has provided a large environmental service to the global climate, then the net carbon emissions of the Amazon nations—Brazil, Bolivia, Colombia, Ecuador, French Guyana, Guyana, Peru, Suriname, Venezuela—may be greatly over-estimated. Typically, national and international assessments simply omit the behaviour of intact forest ecosystems for example while Brazil’s reporting to the UNFCCC includes gross deforestation for all land, carbon removal from the atmosphere is only estimated for managed lands. Second, the renewed emphasis on national reporting of all carbon fluxes following the Paris 2015 climate agreement means that it may well be advantageous for tropical forest nations to examine the behaviour of their old-growth forests extremely carefully. And third, while world leaders have set an ambition of limiting global temperature rise to 1.5 °C above pre-industrial levels, in practice this may only be accomplishable if the biosphere cooperates and provides large net sinks into natural and managed ecosystems worldwide.

Here, we aim to interpret the latest RAINFOR findings in a much more biogeographically, politically, and temporally differentiated way. Our specific objectives are to:

1. Provide a biogeographically differentiated (i.e., region-by-region) assessment of the Amazon forest carbon sink over the last three decades;
2. Provide a politically differentiated (i.e., country-by-country) assessment of the carbon sink over the last three decades.
3. Evaluate the magnitude and trajectory in relation to national anthropogenic carbon emissions (fossil fuels and deforestation) and in relation to estimated land-use related fluxes within Amazonia.

This is the first attempt to evaluate the results on natural forest dynamics from the RAINFOR network in the context of national estimates of fossil-fuel emissions and of land-use change disturbance. The data sources for each of these processes differ greatly. While large anthropogenic and natural disturbance processes are best detected and quantified via remote-sensing methods [e.g., 14, 16], in equatorial forests natural large disturbances and subsequent recovery do not appear to substantially impact large-scale long-term biomass dynamics [17, 26]. Detecting the small changes within mature forests instead typically requires direct tree-by-tree measurements to track the identity, growth, and death of individual trees. Based on such an approach, our analysis here seeks to provide an assessment of the net (“natural”) fluxes as measured in plots to the climate change research and policy communities, by biogeographic and political unit. We thus reanalyse the most up-to-date pan-Amazon dataset of biomass dynamics [10], decade-by-decade, and at the level of biogeographical region and nation state, and compare these fluxes with independent estimates of carbon fluxes from land use change and fossil fuel combustion.

## Methods: summary

Here we summarize our overall approach. Later, in the Additional file ‘Detailed Materials and Methods’, we describe the methodological process in more detail.

We use the plot-by-plot and census-by-census data which were recently analysed to derive overall, Amazon-wide fluxes and trends [10]. These data represent the efforts of more than 100 collaborators in the RAINFOR network (Amazon Forest Inventory Network), using 309 long-term plots in 71 distinct sites across mature Amazon forests. Spatially, we limit our analysis here to the hydrographic Amazon basin plus the contiguous moist forests of the Guiana Shield, so we exclude 11 extra-Amazonian plots in northwest South America presented in Brienen et al. [10]. Temporally, we analyse for three successive decades, the 1980’s, 1990’s, and 2000’s.

We analyse the behaviour of these structurally mature, “old-growth” forest sites in three ways, reporting always our estimates of the net biomass carbon balance together with its estimated uncertainty derived from these plot measurements. We thus estimate the net sink firstly by time across the Amazon, then by biogeographical region across the Amazon, and finally by nation (and by time) across the Amazon. For all the time-differentiated analyses, for simplicity we break down the results by decadal units. For the biogeographically-differentiated analyses we follow a recent approach [19] that divided the lowland tropical forests of South America into five different regions based on biogeographic and biogeochemical evidence to take account of known major ecosystem discontinuities within the region (see Additional file 1: Fig. S1). For our national analyses, we used the biogeographically-based estimates of mean and uncertainty of carbon balance in each region to estimate the area-weighted mean mature Amazon forest carbon balance per country, based on the area of forest represented in each biogeographical region in each nation.

For all these analyses we rely on best estimates of mature forest area as mapped for each country for the year 2000 in the Global Land Cover product [8]. These values were projected forward in time to 2011 and back in time to 1980, by deriving estimates of annualized change rates in Amazon forest area for each country from available sources (see “Methods” section). GLC 2000 land cover class area uncertainties are not available for South American countries, but to provide an alternative and very conservative lower bound to the sink estimates, we repeated all the above analyses using the ‘intact forest landscape’ (IFL) product [45], which, excluding all landscapes which may have direct human impacts, defines IFLs as unbroken expanses of natural ecosystems within areas of current forest extent, without signs of significant human activity, and having an area of at least 500 km^2^ [45].

To compare with the fossil fuel emissions we use a global compilation of national data reported by CDIAC [9]. To estimate deforestation-related emissions, a number of alternative sources are available but no single source provides year-by-year estimates of deforestation-based carbon emissions for all Amazon countries throughout. We therefore developed a hybrid approach, described in “Methods” section, identifying preferred sources based primarily on satellite-based analyses with explicit methodologies [e.g., 46, 53] over nationally compiled statistics [e.g., 18], where possible accounting for estimated non-uniform density of carbon in forests across the Amazon. We also explored an alternative source [25] to assess whether the deforestation estimate we used was likely to be conservative or not, for the period and location for which a direct comparison of estates is possible (2001–2010 Amazon forests).

Finally, for other land-use changes—including fragmentation and edge effects, logging, fire, secondary re-growth and subsequent disturbance—information is much less systematically available through time and across nations, and measurement uncertainties are greater. Given the measurement difficulties and the uneven coverage of available estimates we do not attempt to derive time trends in these processes, and we make a number of necessarily simplifying assumptions (see “Methods” section). Where appropriate we add independent uncertainties in quadrature [e.g., 3], and use a conversion factor of 0.47 to derive the carbon content in tropical biomass [1].

## Results

**Fig. 1:**
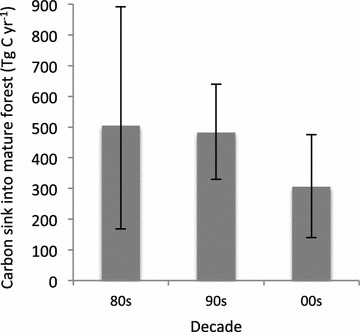
Estimated carbon sink into mature forest biomass in the Amazon basin for each of the three decades since 1980. *Error bars* show 95% confidence intervals

Across the Amazon basin there has been a significant, sustained, but declining net carbon sink into mature forest biomass (Fig. 1). Decade-by-decade, Amazon forests gained biomass at a similar rate during the 1980’s and the 1990’s, at about 500 Tg C per year, although the better sampling in the 1990’s results in much greater confidence in the magnitude of the sink during the 1990’s than the 1980’s (see error bars in Fig. 1). The sink slowed by more than a third during the first decade of the twenty-first century, to ca. 300 Tg C per year. This decline has been caused principally by a weakening of the sink on a per-hectare basis, and less so by the decline in forest area per se. Thus, the net gain in carbon in above-ground forest biomass declined more than 30%, from 0.37 Mg C ha^−1^ yr^−1^ in the 1980’s and 1990’s, to 0.24 Mg C ha^−1^ yr^−1^ in the 2000’s, while total forest area declined less than 10% from an estimated 639 × 10^6^ ha in 1985 to 590 × 10^6^ ha by 2005.

The sink has been widely distributed and not driven by forests in one particular region (see Additional file 1: Table S1a). When divided into five regions based on large-scale geographic and biogeochemical divisions, individual plots in all five regions (Brazilian Shield, Guiana Shield, Upper Amazonia, South-West Amazonia, and East-Central Amazonia) have gained significantly. Among regions the long-term mean estimated gain varied relatively little, from a low of 58 Tg C per year in East-Central Amazonia, to a high of 123 Tg C per year in Brazilian shield (Additional file 1: Table S1b).

When results are broken down into biogeographic regions (see Additional file 1: Fig S1) and decade, the smaller sample sizes available imply reduced confidence in each individual combination of region by time period. Nevertheless, for each of the five regions in each of the three decades (i.e., for all 15 possible space–time combinations) the estimated mean rate of biomass change has been positive (Additional file 1: Table S1b). In 11 of these 15 possible combinations the lower confidence interval was also greater than zero, including for each of the five regions during the 1990’s. While the results show how widespread and persistent the sink has been, the overall decline during the latest decade was not recorded everywhere. Rather, the decline has been sharp in Southwest Amazonia and the Brazilian Shield while in other regions it is not evident.

**Fig. 2:**
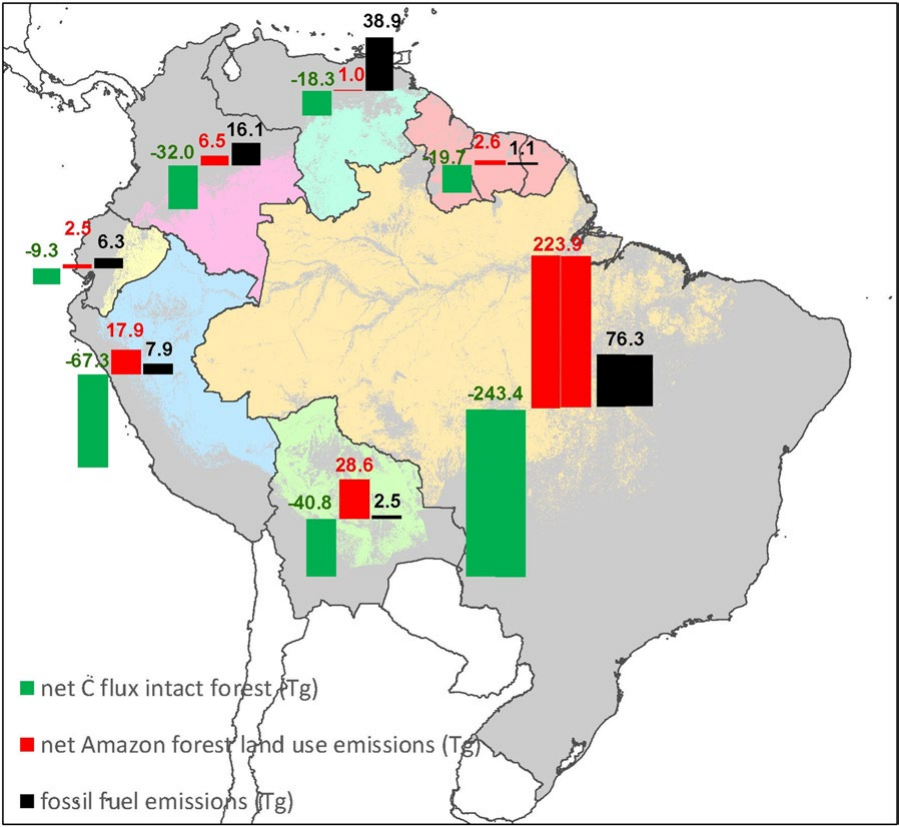
Estimated Amazon carbon fluxes 1980–2010. For each nation three fluxes are represented: the net C flux mature forests (*green* and negative), the net fluxes from deforestation, i.e., losses from deforestation and degradation minus gains from regrowth (*red* and positive), and fossil fuel emissions (*black* and positive). Units are in Tg carbon per year (=10^12^ g C yr^−1^)

Over the whole period, the ground measurements suggest that for each of the nine Amazon nations that mature Amazon forests have provided a net carbon sink, ranging from 4 Tg C per year in the smallest country (French Guiana) to 243 Tg C per year in the largest (Brazil) (see Additional file 1: Table S2). The estimated Amazon-wide forest biomass carbon sink between 1980 and 2010 (430, [213, 669] Tg C yr^−1^) has greatly exceeded the combined emissions from fossil fuel combustion (149 [131, 167] Tg C yr^−1^) for the nine Amazon nations (Fig. 2). This holds also on a national basis for every country except Venezuela. Since the turn of the millennium, the carbon sink has declined while fossil fuel emissions have increased in most South American nations, but the former is still likely to have exceeded the latter (306 (140, 476) vs. 180 Tg C (167, 193)).

As well as fossil fuel combustion, land-use changes in Amazonia have been substantial sources of carbon to the atmosphere. The 1980–2010 combined estimated flux from fossil fuel combustion, and Amazon deforestation, degradation, and fragmentation averaged 431 (326, 538) Tg C, a value which has been remarkably steady, composed of a generally declining land-use component and a generally increasing fossil field component (Table 1). Overall across the three decades, the mature forest sink has approximately mitigated these sources. Note that we estimate a net flux of just 1 Tg, remarkably close to zero, but in the latest decade the combined sources exceeded the mature forest sink for the first time in the record. Alternatively, if we assume conservatively that only the ‘intact forest landscapes’ have contributed sinks and that other mature forests were carbon–neutral, we estimate a somewhat smaller total, with the intact forest sink declining from 342 Tg C in the 1980s to 236 Tg C in the 2000s (Additional file 1: Table S3). Even under this conservative scenario, the forest sink considerably outweighs the fossil fuel emissions of the Amazon nations (Additional file 1: Fig. S2). Finally, for the directly comparable period (2001–2010), Amazon deforestation emissions as estimated from the online Global Forest Watch source average a total of 161 Tg C per year, while our PRODES-based estimate suggests total emissions of 201 Tg C per year in this decade.

**Table 1:** Net C fluxes for the Amazon basin 1980–2009.9, displayed decade by decade

Period	Mature forest Sink	Land use change	Fossil fuel emissions	Net flux
1980–1989.9	−504.4	317.9	105.2	−81.3
1990–1999.9	−482.1	271.7	139.5	−70.8
2000–2009.9	−305.9	275.4	180.0	149.5
1980–2009.9	−430.8	282.9	149.0	1.1

Fluxes are divided into carbon uptake by mature forests, the fossil fuel emissions, fluxes due to land use change and the resulting net flux. Land use change fluxes include emissions resulting from deforestation and forest degradation, and estimate for regrowth. Negative signs indicate removal of carbon from atmosphere, and positive signs indicate net C fluxes from land to the atmosphere. Units are in Tg carbon per year (=10^12^ g C yr^−1^)

## Discussion

This is the first attempt to estimate the ecosystem service of carbon sequestration in mature forests in Amazonia on a long-term regional and national basis. The results suggest that, at least since 1980, the average annual carbon sink into mature forests of the Amazon nations has been at least twice the magnitude of carbon emissions from the same nations’ burning of fossil fuels. Moreover, for every country except for Venezuela the net carbon uptake into mature Amazon forests has exceeded Amazon nations’ total fossil fuel emissions. For most nations the uptake has also exceeded the combined emissions due to fossil fuels and Amazon deforestation, degradation, and fragmentation. Despite lack of knowledge on forest area uncertainties the only comparison with an independent product for land cover class indicates that the GLC product is conservative for forest area in Colombia [22], suggesting that our mature forest sink estimate may be conservative. Further, since our PRODES-based deforestation-related carbon emission estimate exceeded by one fifth a comparable estimate derived from Global Forest Watch, it is possible our anthropogenic CO_2_ emissions estimation methodology may over-estimate the deforestation source, further supporting the conclusion that natural forest sinks in Amazon have compensated for anthropogenic emissions. The mature forest sink is about 30% smaller if we alternatively assume that the only sinks are located in unbroken and expanses of natural forests of at least 500 km^2^ (‘intact forest landscapes’). This represents an extremely conservative and unlikely scenario. In fact, at least half the mature forest plots assembled are located outside these IFLs, including the longest-monitored plots (in Venezuela) and clusters with large, net sinks in Colombia, Ecuador, Peru, and Venezuela.

Thus, not only are the stocks of carbon in Amazon forests very large (exceeding 100 Pg in above- and below-ground biomass, e.g., [40]), but Amazon nations have also contributed to mitigating climate change via net carbon sequestration. The strength of this ecosystem service and its spatial and temporal pattern have implications both for understanding its possible ecological drivers, and for the effective management and conservation of tropical forests in the era of anthropogenic climate change. We first discuss the ecological implications, before addressing the wider implications.

Our analysis shows that the net sink for atmospheric carbon into mature Amazon forests has been an ecologically and geographically ubiquitous pattern. Thus, in all five regions defined a priori on biogeographic and biogeochemical criteria, the sink has been sustained for decades. The ecology and physical geography of these regions differ greatly. For example, while the forests in Southern and Southwestern Amazonia have similar rates of wood productivity as those in the Guiana Shield [34], they typically contain just half the biomass [39], have almost completely different species and phylogenetic composition [32, 55] and greater diversity [54]. Trees in the south and southwest also die at twice the rate of those in the north-east [38], due largely to the strongly divergent geomorphology and soil physical and nutritional conditions [47, 48]. The consistency and long-term persistence of a carbon sink across such different forests indicates that the main driving mechanism is also ubiquitous and long-term. Our findings that the Amazon sink has been geographically widespread and persistent are also consistent with the larger tropical and global picture. Thus, there is compelling evidence from several measurement streams to show that the terrestrial ecosystem sink is persistent and large [e.g., 7, 37] and that most of this has been into forests including in the tropics [e.g., 40]. Together with basic expectations from theory and observations about the ecophysiological impact of increasing atmospheric CO_2_ [e.g., 21], this spatial and temporal persistence implies that stimulation of tree growth by increasing carbon dioxide is at least partly responsible (cf. [52]. The fact that the sink has recently weakened only in the south and south-west, which are *also* the only regions which have experienced an increase in dry season intensity [29], is also instructive. This suggests that the recent Amazon droughts have exerted large-scale but not basin-wide influence. And, so far at least, while these droughts have reversed the carbon sink during individual drought years such as 2005 and 2010 [20, 24, 41], they have not yet done so on a sustained basis.

The findings also have several implications for Amazon forest management and policy. First and most obviously, from a historical perspective, if all of Amazonia has provided a carbon sink environmental service to the global climate, then it follows that the net carbon emissions of the Amazon nations—Brazil, Bolivia, Colombia, Ecuador, French Guyana, Guyana, Peru, Suriname and Venezuela—must have been seriously over-estimated in all assessments that omit to consider the carbon balance of mature forest ecosystems. While many northern countries include the carbon balance of their intact forest lands (which also tend to be a net sink e.g., [40]) in their reporting to the UNFCCC, Amazon countries have simply excluded carbon dynamics in old growth forests in their reporting.

Second, while there is rightly increasing emphasis on managing secondary forests for their carbon sink potential [e.g., 12] our results suggest that at a national level tropical secondary forests may not in fact provide the largest forest sinks. While potential maximum rates of carbon sequestration per unit area are high in secondary forests for several decades following clearance [44], in Amazonia landscapes characterized by a mosaic of cropland, degraded and secondary forests are also at greatly enhanced risk of fire [5] or other degradation and deforestation processes [e.g., 2]. This, together with the large area that remains of structurally mature forest in Amazonia, means that the total carbon sequestration provided by mature forests has almost certainly been much greater than the net sequestration from secondary systems. Whether it continues to be so or not is of course unknown, but our estimates here are that in the decade since 2000 mature Amazon forests contributed 306 (140, 476) Tg C every year, while secondary forest recovery contributed 60 (34, 84) Mg C. The latter estimate is less than 30% of the potential estimated total annual sink for secondary forests in the neotropics if all were left to regrow (ca. 8 Pg over 40 years, [12]), but it is based on one high-resolution analysis [6]. Clearly, a research priority for the future must be to better understand the dynamics of forest carbon emissions in landscapes undergoing rapid land use change, including fragmentation, regrowth, deforestation, and degradation processes.

Third, and consequent on both points above, it remains feasible that in most Amazon nations the land remaining as forest can still provide net carbon sinks well into the future. Via a combination of protection of old-growth forests and some enhanced secondary forest recovery, the potential carbon sequestration benefits of Amazonia for mitigating climate change are strong. The extent to which these climate services are actually realised depends on many factors. While only some of these lie within the control of Amazon nations themselves, the protection of old-growth forests is a matter of national policy. The increased emphasis on national reporting of carbon fluxes following the Paris 2015 climate agreement means that tropical forest nations which protect remaining mature forests and carefully monitor and report the behaviour and subtle changes occurring within them may stand to benefit materially.

## Conclusions

Results from standardised, ground-based monitoring of the growth and death of individual trees have been used to build a picture of the behaviour of mature forests across the Amazon basin since the 1980’s. The picture that emerges is one of forests far from equilibrium, with both growth and mortality rates having risen and with a persistent and geographically very widespread difference between the two that implies a carbon sink into mature forests across the whole region. The net sink has substantially affected the long-term carbon budgets of all nine Amazon nations, exceeding the fossil fuel emissions in eight of them. While fossil fuel emissions have been increasing and the sink has recently weakened in some parts of the basin, mature forests in all nine nations continued to contribute substantial net sequestration of carbon over the most recent decade. Overall, in most tropical countries emissions and removals by forests dominate national net C flux profiles. If these developing countries are to contribute to global climate change mitigation, it is forests that will need to be managed to both increase removals and reduce emissions.

Whether or not Amazon nations will in turn benefit from this global ecosystem service in coming years is unclear. To achieve such benefits requires a better understanding of how carbon dioxide, climate and other ‘indirect’ anthropogenic factors are actually affecting old-growth forests. This in turn requires a significant increase in the level of investment in tropical forest monitoring, combining ground-based and remotely-sensing techniques, especially so in protected areas. At both national and global levels, a step-change in the magnitude and coordination of such work is needed in order to track the behaviour of these uniquely valuable ecosystems.

## Additional file

**Additional file 1.** Detailed materials and methods; supplementary tables and figures.

## Abbreviations

GLC2000: Global Land Cover 2000
IFLs: intact forest landscapes
GHGs: greenhouse gases
PRODES: Projeto Prodes Monitoramento da Floresta Amazônica Brasileira por Satélite
RAINFOR: Amazon Forest Inventory Network (Red Amazónica de Inventarios Forestales)
