# Supplementary material for: Carbon uptake by mature Amazon forests has mitigated Amazon nations’ carbon emissions
Source: Carbon Balance Manag. 2017 Feb 15;12:1. doi: 10.1186/s13021-016-0069-2 (PMC5285296; doi:10.1186/s13021-016-0069-2)

**Carbon uptake by mature Amazon forests has mitigated Amazon nations’ carbon emissions**

**O.L. Phillips and R. Brienen**

**Additional file**

**Detailed Materials and Methods**

**Old-growth forest C fluxes**

To estimate carbon fluxes into mature old growth forests, we used net biomass change data from inventory plots from the RAINFOR network and from published plot data as published by Brienen et al. 2015, excluding only 11 plots from extra-Amazonian north-west South America. This dataset includes terra firme, floodplain, white sand and swamp forests from lowland tropical areas of Amazonia and contiguous Guiana Shield forests (below 1,500 m above sea level) that receive at least 1,000 mm of rainfall annually. Immature or open forests, and those known to have had anthropogenic disturbances owing to fire or selective logging, were excluded. The inventory plots are geographically dispersed throughout the Amazon Basin (c.f. Brienen et al. 2015, Extended Data Fig. 1).

For each plot all stems greater than 100 mm were identified and their diameter measured at breast height, defined as 1.3 m from the base of the stem. For non-cylindrical stems owing to buttresses or other deformities the point of measurement (POM) is raised to ca. 50 cm above deformities or buttresses, If these changes in POM were made we recorded both the diameter at the original POM and the new POM and used the approach described in Brienen et al. (2015) and detailed in Talbot et al. (2014) to calculate a diameter growth series from the two disjoint series. Different approaches for dealing with these POM changes give slightly different outcome in terms of the magnitude of the biomass sink, but in all cases lead to significant biomass gains.

We estimated the net biomass change for each census interval as the difference between standing biomass at the end of the census period and the beginning of the interval divided by the census length. To calculate biomass, we used allometric equations described previously (Feldpausch et al. 2012) to convert tree diameter, height and wood density to woody biomass or carbon. Tree height was estimated using established diameter height equations that vary between the different regions of the Amazon (see Feldpausch et al. 2012). Wood density values were extracted from a global wood density database (http://datadryad.org/handle/10255/dryad.235; Chave et al. 2009). In our calculations for biomass, we also included biomass components that were not directly measured, assuming that these pools responded proportionally to the measured above ground biomass in trees bigger than 10 cm in diameter. Based on destructive measurements of stand biomass in central Amazonia (see Phillips et al. 2008), we added an additional fraction of ~9.9% of the measured above ground biomass for lianas and trees smaller than 100 mm in diameter represent, and assumed that below ground biomass is a fraction of ~37% of above ground biomass. We used a conversion factor of 0.47 to convert biomass to carbon, following IPCC guidelines (Aalde et al., 2006).

To account for differences in the monitoring effort allocated to individual plots we use the same area- and time-weighting procedures as described and evaluated by Brienen et al. 2015. Likewise, for analysis purposes, plots smaller than 0.5 ha that were within 1 km or less of one another were merged, to give a total of 267 ‘sample units’. The mean size across all sample units was 1.26 ha, and the mean total monitoring period was 15.8 years. In total, the study monitored 337 ha for a combined total of 4,438 ha years, involving more than 787,000 tree measurements on around 175,000 individual trees larger than 10 cm diameter. In our calculations we do not attempt to construct local trends for each individual plot (unlike Brienen et al. 2015), as our objective here is not to derive estimates of trends in biomass dynamics and balance, but rather to provide the best estimate of net change in each period using all plots available in each period and each region.

To scale up inventory estimates of carbon change to country, region and basin-wide estimates of carbon fluxes, we used forest area estimates from the Global Land Cover 2000 dataset (Bartholomé & Belward 2005) within the hydrographic Amazon basin for Brazil, Bolivia, Peru, and Colombia. For Venezuela, Guyana, Suriname, and French Guiana, we include the contiguous moist forests of the Guiana Shield. This definition is designed to match the extent of the originally contiguous Amazonian biogeographic region, and also corresponds for Brazil, Bolivia, Colombia, Ecuador and Peru to the definitions used by Song et al. (2015) in their detailed analyses of deforestation rates. Within this domain we divide the Amazon into five biogeographic regions, the Brazilian shield, the south-west Amazon, the central-east Amazon, the Guyana shield, and the central-west Amazon following Feldpausch et al. 2011 and Brienen et al. 2015 and mapped in SI Fig 1. Total carbon fluxes are obtained by multiplying the mean net change per hectare for each biogeographic area (see SI Table 1a) times the area of mature forest within each region from Global Land Cover 2000 dataset, or the ‘Intact Forest Landscape’ (IFL) product (Potapov et al. 2008). Fluxes for individual countries were calculated by summing the fluxes into mature forests within each biogeographic region within each country. We accounted in these calculations for changes in forest area using annualized deforestation rates by country as described in the following section. Based on the forest area from GLC or IFL in the year 2000 we calculated the forest area for each country and biogeographic region for each year since 1980. Decadal scale fluxes for mature forests were based on forest area at the middle of the decade.

We note that the IFL product provides a very conservative lower bound on mature forest area, but it would be desirable too to assess uncertainties on the GLC2000 Land Cover product. These do not appear to be available, at either country or region-wide level. Some evaluations of this and related products exist but typically involve assessing pixel-by-pixel levels of agreement/disagreement between products and are rarely differentiated for regions relevant for us (e.g. Friedl et al. 2011, Fritz et al. 2011). For the present purposes, pixel level uncertainties are essentially irrelevant (we care about the aggregate, national level uncertainty), and in any case we require product validation against the ground reality, rather than against alternate remote sensing modelled products. Within these constraints we explored the likely potential impact of uncertainties in the GLC product. We conclude that it is unlikely to result in an overestimate of the mature forest total sink. Thus: (1) We explored adding random error for each country level, and found that such country level random error makes only a marginal difference to the overall, integrated error of the Amazon-level sink, since these fractional uncertainties are added in quadrature when scaled to the Amazon. For instance, even including a hypothetical 50% uncertainty on the total forest area value for each country (either positive or negative) results in only a 10% increase in uncertainty in the total Amazon carbon sink, because the country-level errors tend to cancel. (2) Alternatively, we could chose to specify Amazon level error on the Amazon total forest area estimate, in which case the error on the integrated Amazon level sink would be greater. Thus the question is whether there is a systematic large-scale bias in the GLC approach. We are not aware of any publications which show this. However, since GLC is based on a 20-m resolution spectrometer (VEGETATION, on-board the SPOT satellite), it might be expected to yield more precise and less biased estimates than coarse-resolution sources. In the only inter-assessment comparison that we could find (Fritz et al. 2008 – comparing with a lower-resolution MODIS product), this global analysis shows that for Colombian forests that the GLC forest classes estimate *less* forest area as compared to MODIS assessments (MODIS v.5 (IGBP)). Therefore while we lack forest area uncertainty estimates on a country by country and year by year basis, there is some evidence that the area estimates that we used are conservative, and therefore that our intact forest carbon sink estimates are also conservative.

**Deforestation-based carbon emissions**

A number of alternative sources are available but no single source provides year-by-year estimates of deforestation-based carbon emissions for all Amazon countries through the whole period. In identifying the preferred sources for our study we used the following criteria: 1. prefer more recent sources where available, over older sources; 2. prefer satellite-based analyses over national reporting statistics (e.g. FRA); 3. prefer sources that attempt to also account for the non-uniform density of carbon in forests across the Amazon.

In general, for data sources for deforestation estimates we therefore used for Brazilian Legal Amazonia the 1988-2013 PRODES dataset, produced by the Brazilian Space Agency (INPE). This is widely recognised as providing a long methodologically consistent analysis, and because this is the most important Amazon nation other authors have simply scaled the PRODES numbers to all of South American tropical forests (e.g. Gloor et al. 2012). Recently, Song et al. (2015) have derived annual deforestation indicators since 2000 using the Moderate Resolution Imaging Spectroradiometer Vegetation Continuous Fields (MODIS VCF) product, calibrated these with Landsat data to generate accurate deforestation rates, and then combined these with a spatially explicit biomass estimates to calculate committed annual carbon emissions. This being an alternative and somewhat complementary analysis to PRODES, and available for other Amazon nations, we used this source for estimating deforestation C emissions for Brazil, Bolivia, Colombia, and Peru for 2000-2010, the four nations responsible for ≈99% of Amazon losses. For estimating emissions from the remaining minor contributors (Ecuador, French Guiana, Guyana, Suriname, and Venezuela) we used analysis based on Hansen et al. 2013 and Global Forest Watch to derive an estimate for area-based losses in 2001-2011. To convert these area losses to carbon emissions estimates, we applied the mean carbon biomass density in Amazon forest lost in 2000-2010 (Song et al. 2015).

For the period pre-2001 there is no single, satellite-based analytical source for Amazon carbon losses. But for Brazil, responsible for ca. 80% of deforestation emissions, a consistent satellite-based deforestation sequence is available from PRODES (2015) for our entire 1980 to 2011 window. PRODES provides annualised estimates of loss of newly cleared land in Amazonian Brazil, in area terms but not in carbon terms. To estimate Brazilian forest carbon losses for each year pre-2001 we used the PRODES area baseline, and scaled each years area losses by the mean per area carbon density in Amazon forest lost in 2000-2010 (from Song et al. 2015). To estimate non-Brazilian carbon losses pre-2001 we applied the ratio of total area lost for each nation relative to Brazil in the 2000-2010 period (from Song et al. 2015 and Hansen et al. 2013) to the pre-2001 area losses derived from the PRODES series. Carbon density in those lost forests was estimated as the mean carbon density for Amazon forest lost in 2000-2010 as before.

We follow Song et al. in allocating an uncertainty range of +38% to the carbon emission rate estimates, based on their estimates of deforestation area uncertainty associated with the MODIS VCF and Landsat samples, and those associated with biomass distribution. Note that Song et al. (2015) conclude that only one third of the emission uncertainties are area-related inherited from the deforestation map, while two thirds are from uncertainties in carbon density in the biomass map, so the greatest reductions in uncertainty concerning the magnitude of carbon fluxes due to Amazon deforestation are unlikely to come from more precise estimates of deforestation (welcome as they are), but with more spatially accurate estimates of the distribution of above-ground biomass across the forested and previously-forested landscape.

We also explored an alternative source (Global Forest Watch, available since the year 2000) to assess whether the deforestation estimate we used was likely to be conservative or not, for the period and location for which a direct comparison of estates is possible (2001-2010 Amazon forests). The GFW-based emission estimate averages a total of 161 Tg C per year, while the PRODES-based estimate we used suggests total emissions of 201 Tg C per year in this decade. Thus we conclude that, our anthropogenic CO2 emissions estimation methodology is more likely than not to over-estimate the deforestation source, further supporting our central conclusion that natural forest sinks in Amazon have compensated for anthropogenic emissions.

For **other land-use changes** information is less systematically available through time and across nations, is more heavily dependent on local contexts, and is subject to greater measurement uncertainties. The principle relevant processes include fragmentation and edge effects, logging, fire, and re-growth. Given the measurement difficulties and the highly uneven coverage of available estimates we do not attempt to derive time trends in these processes, and we make a number of necessarily simplifying assumptions. For fragmentation emissions, we use the recent estimate of Pütz et al. (2010), based on long-term analysis of Amazonia using Modis imagery, totalling losses of 599.1 Tg C (+20% uncertainty) over 30 years, 1980-2009, across the whole of Amazonia, at an annual rate of 19.97 Tg C. We allocated this fragmentation flux to each Amazon nation in proportion to their deforestation carbon emissions. For logging-related emissions, we use Asner et al.’s (2010) estimate of ≈80 Tg C yr-1 for Brazilian Amazonia for 1999-2002. We assumed this emission rate throughout the period, and for other nations scaled by the relative forest area. For secondary forest regrowth we use Asner et al. estimate that secondary regrowth provided an 18% offset against total gross emissions, which yields a similar estimate to Houghton et al. (2000) of ≈60 Tg C yr-1 for Brazilian Amazonia for the late 1990’s. Uncertainty in the logging- and regrowth estimates was estimated by adding, in quadrature, the uncertainties in forest area and carbon density (Song et al. 2015) and fragmentation (Putz et al. 2010).

In summing the land-use change flux estimates we note that there may be some double-counting of LUCC carbon losses when summed over many subsequent years. For example, forest frontiers that are selectively logged, fragmented, or otherwise degraded are at greater risk of becoming deforested subsequently. In particular large areas of southern Amazonia that were selectively logged in recent decades are now under cultivation (e.g., Brown et al. 2005). For this reason it is possible that our approach may result in overestimating net fluxes due to land use change processes.

**Fossil Fuel and Cement emissions**

We used the national emissions inventory data published by Boden et al. (2013). These provide annualised emissions estimates for the sum of geological carbon emissions (solid, liquid, and gas fossil fuels, and emissions from cement manufacture). We excluded bunker fuel emissions from international shipping, as this is a relatively small source and poorly attributed to nations for some of the record. We adopt the Andres et al. (2014) estimate that the independent, national-level 2 sigma uncertainties for these fluxes are 12.1% of annual values, and again when summing fluxes across nations we follow convention (e.g., Aragao et al. 2009) in adding independent uncertainties in quadrature.

**Detailed Methods References**

Andres RJ, Boden TA, Higdon D. A new evaluation of the uncertainty associated with CDIAC estimates of fossil fuel carbon dioxide emission. *Tellus B*. 2014 Jul 14;66.

Aragão LE, Malhi Y, Metcalfe DB, Silva-Espejo JE, Jiménez E, Navarrete D, Almeida S, Costa AC, Salinas N, Phillips OL, Anderson LO. Above-and below-ground net primary productivity across ten Amazonian forests on contrasting soils. *Biogeosciences*. 2009 Dec 1;6(12):2759-78..

Asner GP, Powell GV, Mascaro J, Knapp DE, Clark JK, Jacobson J, Kennedy-Bowdoin T, Balaji A, Paez-Acosta G, Victoria E, Secada L. High-resolution forest carbon stocks and emissions in the Amazon. *Proceedings of the National Academy of Sciences*. 2010 Sep 21;107(38):16738-42.

Bartholomé E & Belward A. GLC2000: a new approach to global land cover mapping from Earth observation data. Int. J. Remote Sens. 2005. 26:1959–1977.

Boden TA, Marland G, Andres RJ. 2013. Global, Regional, and National Fossil-Fuel CO2 Emissions. Carbon Dioxide Information Analysis Center, Oak Ridge National Laboratory, U.S. Department of Energy, Oak Ridge, Tenn., U.S.A. DOI: 10.3334/CDIAC/00001_V2013*. downloaded 8 September 2015*

Brown JC, Koeppe M, Coles B, Price KP. Soybean production and conversion of tropical forest in the Brazilian Amazon: The case of Vilhena, Rondonia. *AMBIO: A Journal of the Human Environment*. 2005 Aug;34(6):462-9.

Chave J, Coomes D, Jansen S, Lewis SL, Swenson NG, Zanne AE. Towards a worldwide wood economics spectrum. *Ecology Letters*. 2009 Apr 1;12(4):351-66.

Feldpausch TR, Lloyd J, Lewis SL, Brienen RJ, Gloor M, Monteagudo Mendoza A, Lopez-Gonzalez G, Banin L, Abu Salim K, Affum-Baffoe K, Alexiades M. Tree height integrated into pantropical forest biomass estimates. *Biogeosciences*. 2012 Aug 27:3381-403.

Friedl MA, Sulla-Menashe D, Tan B, Schneider A, Ramankutty N, Sibley A, Huang X. MODIS Collection 5 global land cover: algorithm refinements and characterization of new datasets *Remote Sensing of Environment.* 2010 114 168–82.

Fritz S, See L, McCallum I, Schill C, Obersteiner M, Van der Velde M, Boettcher H, Havlík P, Achard F. Highlighting continued uncertainty in global land cover maps for the user community. *Environmental Research Letters*. 2011 4:044005.

Global Forest Watch. 2014. World Resources Institute. Accessed on 21 November 2016. [www.globalforestwatch.org](http://www.globalforestwatch.org/).

Hansen MC, Potapov PV, Moore R, Hancher M, Turubanova SA, Tyukavina A, Thau D, Stehman SV, Goetz SJ, Loveland TR, Kommareddy A. High-resolution global maps of 21st-century forest cover change*. Science*. 2013 Nov 15;342(6160):850-3.

Data sourced via: htttp://mongabay-images.s3.amazonaws.com/gfw/forest-loss-non-brazilian-amazon-2001-2012.jpg

Houghton RA, Skole DL, Nobre CA, Hackler JL, Lawrence KT, Chomentowski WH. Annual fluxes of carbon from deforestation and regrowth in the Brazilian Amazon. *Nature*. 2000 Jan 20;403(6767):301-4.

Lopez‐Gonzalez G, Lewis SL, Burkitt M, Phillips OL. ForestPlots. net: a web application and research tool to manage and analyse tropical forest plot data. *Journal of Vegetation Science*. 2011 Aug 1;22(4):610-3.

Numata I, Cochrane MA, Souza Jr CM, Sales MH. Carbon emissions from deforestation and forest fragmentation in the Brazilian Amazon*. Environmental Research Letters*. 2011 Oct 10;6(4):044003.

Phillips OL, Baker T, Brienen R & Feldpausch T. RAINFOR field manual for plot establishment and remeasurement. http://.rainfor.org/upload/ManualsEnglish/RAINFOR_field_manual_version_June_2009_ENG.pdf (2010).

Phillips OL, Lewis SL, Baker TR, Chao KJ, Higuchi N. The changing Amazon forest. *Philosophical Transactions of the Royal Society B: Biological Sciences*. 2008 May 27;363(1498):1819-27.

Potapov P, Yaroshenko A, Turubanova S, Dubinin M, Laestadius L, Thies C, Aksenov D, Egorov A, Yesipova Y, Glushkov I, Karpachevskiy M. Mapping the world’s intact forest landscapes by remote sensing. *Ecology and Society*. 2008 Dec 1;13(2):51.

PRODES: Brazilian government Deforestation estimates based on remote sensing: http://www.obt.inpe.br/prodes, 2015.

Pütz S, Groeneveld J, Henle K, Knogge C, Martensen AC, Metz M, Metzger JP, Ribeiro MC, de Paula MD, Huth A. Long-term carbon loss in fragmented Neotropical forests. *Nature Communications*. 2014 Oct 7;5.

Song XP, Huang C, Saatchi SS, Hansen MC, Townshend JR. Annual carbon emissions from deforestation in the Amazon Basin between 2000 and 2010. *PloS one*. 2015 May 7;10(5):e0126754.

Talbot J. *et al.* Methods to estimate aboveground wood productivity from long-term forest inventory plots. Forest Ecology and Management 2014, 320:30-38.

**Additional file Table S1a. Forest cover, number of permanent monitoring plots and sampling area, and mean above ground** **carbon change as measured in RAINFOR plots in each of the five climate and geomorphological regions in the Amazon basin.** Negative signs indicate removal of carbon from atmosphere by the forest, i.e. a net carbon sink into mature forest. The mean and uncertainty in per-hectare above ground carbon change for the whole basin are weighted by the forest cover area in each biogeographic region. See SI Fig. 1 for distribution of regions.

| Region | Forest cover in 2000 (x10^6^ ha) | N plots | Total sample area  (ha) | Hectare* years monitoring | Mean carbon change  (Mg C ha^-1^ yr^-1^) | | CI | |
| --- | --- | --- | --- | --- | --- | --- | --- | --- |
| Brazilian Shield | 161.8 | 25 | 25.7 | 288.9 | -0.277 | +0.045, -0.599 | |  |
| South west Amazon | 83.2 | 52 | 57.0 | 891.5 | -0.444 | -0.291, -0.651 | |  |
| Central east Amazon | 106.3 | 132 | 113.0 | 1264.6 | -0.373 | -0.230, -0.522 | |  |
| Guyana Shield | 151.1 | 61 | 83.4 | 1435.8 | -0.412 | -0.222, -0.663 | |  |
| Central west Amazon | 99.2 | 39 | 58.4 | 557.6 | -0.482 | -0.183, -0.647 | |  |
| Whole Amazon | 601.7 | 309 | 337.5 | 4438.3 | -0.385 | -0.155, -0.617 | |  |

**Additional file Table S1b. Above ground biomass carbon change for the geographical regions of the Amazon basin displayed decade by decade.** Region-wide means and lower and higher confidence intervals in 10^12^ g Carbon per year (Tg C yr^-1^) are shown. Negative signs indicate removal of carbon from atmosphere by the forest, i.e. a net carbon sink into mature forest. Confidence intervals for the whole basin were estimated by adding the uncertainties for each geographic region in quadrature (i.e., square root of the sum of the squares).

| Region | **1980-1989.9** | | **1990-1999.9** | | **2000-2009.9** | | **Full period** | |
| --- | --- | --- | --- | --- | --- | --- | --- | --- |
| Brazilian Shield | -177 | (+8,-416) | -141 | (68,212) | -50 | (+48,-146) | -123 | (-4,-258) |
| South west Amazon | -87 | (-30,-116) | -103 | (83,123) | -39 | (-7,-67) | -76 | (-40,-102) |
| Central east Amazon | -41 | (+56,-128) | -76 | (40,120) | -57 | (-30,-84) | -58 | (-5,-111) |
| Guyana Shield | -36 | (+49,-174) | -96 | (35,165) | -98 | (-36,-178) | -77 | (-7,-172) |
| Central west Amazon | -164 | (-100,-206) | -66 | (10,106) | -62 | (-19,-102) | -97 | (-43,-138) |
| Whole Amazon | -504 | (-242,-836) | -482 | (-293,-661) | -306 | (-6, -484) | -431 | (-194, -624) |

**Additional file Table S2a. Net biomass carbon change for the countries of the Amazon basin, 1980-2009.9, displayed decade by decade.** [= the tabular equivalent of Fig 2]. To estimate forest area for each country, we used the 2000 Global Land Cover map to provide fixed estimates of area for 2000, and adjusted area calculations before and after using cumulative deforestation losses and land use change emissions as described in Methods. Negative signs indicate removal of carbon from atmosphere by the forest, i.e. a net carbon sink. Units are 10^12^ g Carbon per year (Tg C yr^-1^).

|  | 1980-1989.9 | |  |  | 1990-1999.9 | |  |  | 2000-2009.9 | | |  | Full period | | |  |
| --- | --- | --- | --- | --- | --- | --- | --- | --- | --- | --- | --- | --- | --- | --- | --- | --- |
| Country | Mature forest Sink | Land use change | Fossil fuel emissions | Net flux | Mature forest Sink | Land use change | Fossil fuel emissions | Net flux | Mature forest Sink | Land use change | Fossil fuel emissions | Net flux | Mature forest Sink | Land use change | Fossil fuel emissions | Net flux |
| Bolivia | -50 | 32 | 1 | -17 | -53 | 28 | 2 | -23 | -19 | 27 | 3 | 11 | -41 | 29 | 3 | -10 |
| Brazil | -280 | 251 | 51 | 22 | -285 | 214 | 71 | 0 | -166 | 221 | 95 | 150 | -243 | 224 | 76 | 57 |
| Colombia | -45 | 7 | 13 | -24 | -26 | 6 | 17 | -3 | -25 | 6 | 16 | -3 | -32 | 6 | 16 | -9 |
| Ecuador | -16 | 3 | 5 | -8 | -6 | 2 | 6 | 2 | -6 | 2 | 8 | 4 | -9 | 3 | 6 | 0 |
| French Guiana | -2 | 1 | 0 | -1 | -5 | 1 | 0 | -4 | -5 | 1 | 0 | -5 | -4 | 1 | 0 | -3 |
| Guyana | -4 | 1 | 0 | -2 | -11 | 1 | 0 | -10 | -12 | 1 | 0 | -10 | -9 | 1 | 0 | -7 |
| Peru | -97 | 21 | 6 | -71 | -64 | 17 | 7 | -40 | -40 | 16 | 10 | -14 | -67 | 18 | 8 | -42 |
| Suriname | -3 | 1 | 0 | -2 | -8 | 1 | 1 | -7 | -9 | 1 | 1 | -7 | -7 | 1 | 1 | -5 |
| Venezuela | -8 | 1 | 28 | 20 | -23 | 1 | 36 | 14 | -24 | 1 | 47 | 24 | -18 | 1 | 39 | 22 |
|  |  |  |  |  |  |  |  |  |  |  |  |  |  |  |  |  |
| Amazon sum | -504 | 318 | 105 | -81 | -482 | 272 | 140 | -71 | -306 | 275 | 180 | 150 | -431 | 283 | 149 | 1 |

**Additional file Table S2b. Confidence intervals for the estimated sink by decade for each country.** Confidence intervals for the whole basin were calculated by adding the uncertainties for each geographic region in quadrature (i.e., square root of the sum of the squares). Negative signs indicate removal of carbon from atmosphere by the forest, i.e. a net carbon sink. Units are 10^12^ g Carbon per year (Tg C yr^-1^).

|  | 1980-1989.9 | | 1990-1999.9 | | 2000-2009.9 | | Full period | | | | |
| --- | --- | --- | --- | --- | --- | --- | --- | --- | --- | --- | --- |
| Country | Intact forest Sink | | Intact forest Sink | | Intact forest Sink | | Intact forest Sink | | | | |
| Bolivia | -50 | (-12,-82) | -53 | (-39,-67) | -19 | (1,-38) | -41 | (-16,-62) | | |  |
| Brazil | -280 | (52,-662) | -285 | (-139,-438) | -166 | (-5,-330) | -243 | (-31,-476) | | |  |
| Colombia | -45 | (-20,-68) | -26 | (-6,-43) | -25 | (-9,-43) | -32 | (-12,-51) | | |  |
| Ecuador | -16 | (-9,-20) | -6 | (-1,-10) | -6 | (-2,-10) | -9 | (-4,-13) | | |  |
| French Guiana | -2 | (3,-9) | -5 | (-2,-9) | -5 | (-2,-10) | -4 | (0,-9) | | |  |
| Guyana | -4 | (6,-20) | -11 | (-4,-19) | -12 | (-4,-21) | -9 | (-1,-20) | | |  |
| Peru | -97 | (-88,-125) | -64 | (-34,-88) | -40 | (-10,-67) | -67 | (-44,-93) | | |  |
| Suriname | -3 | (4,-15) | -8 | (-3,-14) | -9 | (-3,-16) | -7 | (-1,-15) | | |  |
| Venezuela | -8 | (11,-40) | -23 | (-8,-39) | -24 | (-9,-43) | -18 | (-2,-41) | | |  |
|  |  |  |  |  |  |  |  | |  |  |  |
| Amazon sum | -504 | (-242,-836) | -482 | (-293,-661) | -306 | (-6, -484) | -431 | | (-194, -624) |  |  |

**Additional file Table S3. Estimated Amazon carbon fluxes 1980-2010.** As **Additional file** Table S2a, but assuming that only mature forests within ‘Intact Forest Landscapes’ act as carbon sinks. Intact Forest Landscapes are defined as unbroken expanses of natural ecosystems within areas of current forest extent, without signs of significant human activity, and having an area of at least 500 km^2^ (Potapov et al. 2008).

|  | 1980-1989.9 | |  |  | 1990-1999.9 | |  |  | 2000-2009.9 | | |  | Full period | | |  |
| --- | --- | --- | --- | --- | --- | --- | --- | --- | --- | --- | --- | --- | --- | --- | --- | --- |
| Country | Mature forest Sink | Land use change | Fossil fuel emissions | Net flux | Mature forest Sink | Land use change | Fossil fuel emissions | Net flux | Mature forest Sink | Land use change | Fossil fuel emissions | Net flux | Mature forest Sink | Land use change | Fossil fuel emissions | Net flux |
| Bolivia | -22 | 32 | 1 | 12 | -22 | 28 | 2 | 8 | -8 | 27 | 3 | 22 | -17 | 29 | 3 | 14 |
| Brazil | -176 | 251 | 51 | 126 | -193 | 214 | 71 | 92 | -126 | 221 | 95 | 189 | -165 | 224 | 76 | 135 |
| Colombia | -38 | 7 | 13 | -17 | -22 | 6 | 17 | 1 | -22 | 6 | 16 | 1 | -27 | 6 | 16 | -5 |
| Ecuador | -9 | 3 | 5 | -2 | -4 | 2 | 6 | 4 | -4 | 2 | 8 | 6 | -6 | 3 | 6 | 3 |
| French Guiana | -2 | 1 | 0 | -1 | -4 | 1 | 0 | -3 | -4 | 1 | 0 | -4 | -3 | 1 | 0 | -3 |
| Guyana | -3 | 1 | 0 | -2 | -9 | 1 | 0 | -8 | -10 | 1 | 0 | -8 | -8 | 1 | 0 | -6 |
| Peru | -82 | 21 | 6 | -56 | -51 | 17 | 7 | -27 | -56 | 16 | 10 | -30 | 0 | 18 | 8 | 26 |
| Suriname | -3 | 1 | 0 | -1 | -7 | 1 | 1 | -6 | -7 | 1 | 1 | -6 | -6 | 1 | 1 | -4 |
| Venezuela | -7 | 1 | 28 | 22 | -20 | 1 | 36 | 18 | -21 | 1 | 47 | 28 | -16 | 1 | 39 | 24 |
|  |  |  |  |  |  |  |  |  |  |  |  |  |  |  |  |  |
| Amazon sum | -342 | 318 | 105 | 81 | -333 | 272 | 140 | 78 | -236 | 275 | 180 | 220 | -304 | 283 | 149 | 128 |

**Additional file Figure S1. Map of five biogeographic regions distinguished in this study based on a priori defined biogeographic and biogeochemical criteria (see Feldpausch et al. 2011).** The thick black line indicates the border of the Amazon hydrogeographic basin.

**
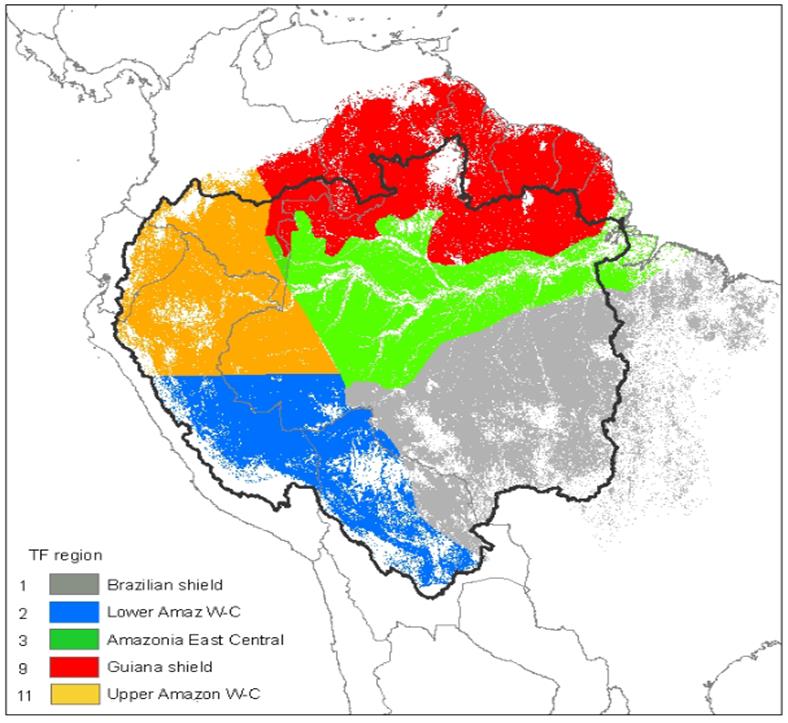
**

**Additional file Figure S2. Estimated Amazon carbon fluxes 1980-2010.** As for Figure 2, but assuming that only mature forests within ‘Intact Forest Landscapes’ act as carbon sinks, and that all other mature forests are carbon-neutral. For each nation three fluxes are represented: the net C flux mature forests (green and negative), the net fluxes from deforestation, i.e., losses from deforestation and degradation minus gains from regrowth (red and positive), and fossil fuel emissions (black and positive). Intact Forest Landscapes are defined as unbroken expanses of natural ecosystems within areas of current forest extent, without signs of significant human activity, and having an area of at least 500 km^2^ (Potapov et al. 2008).


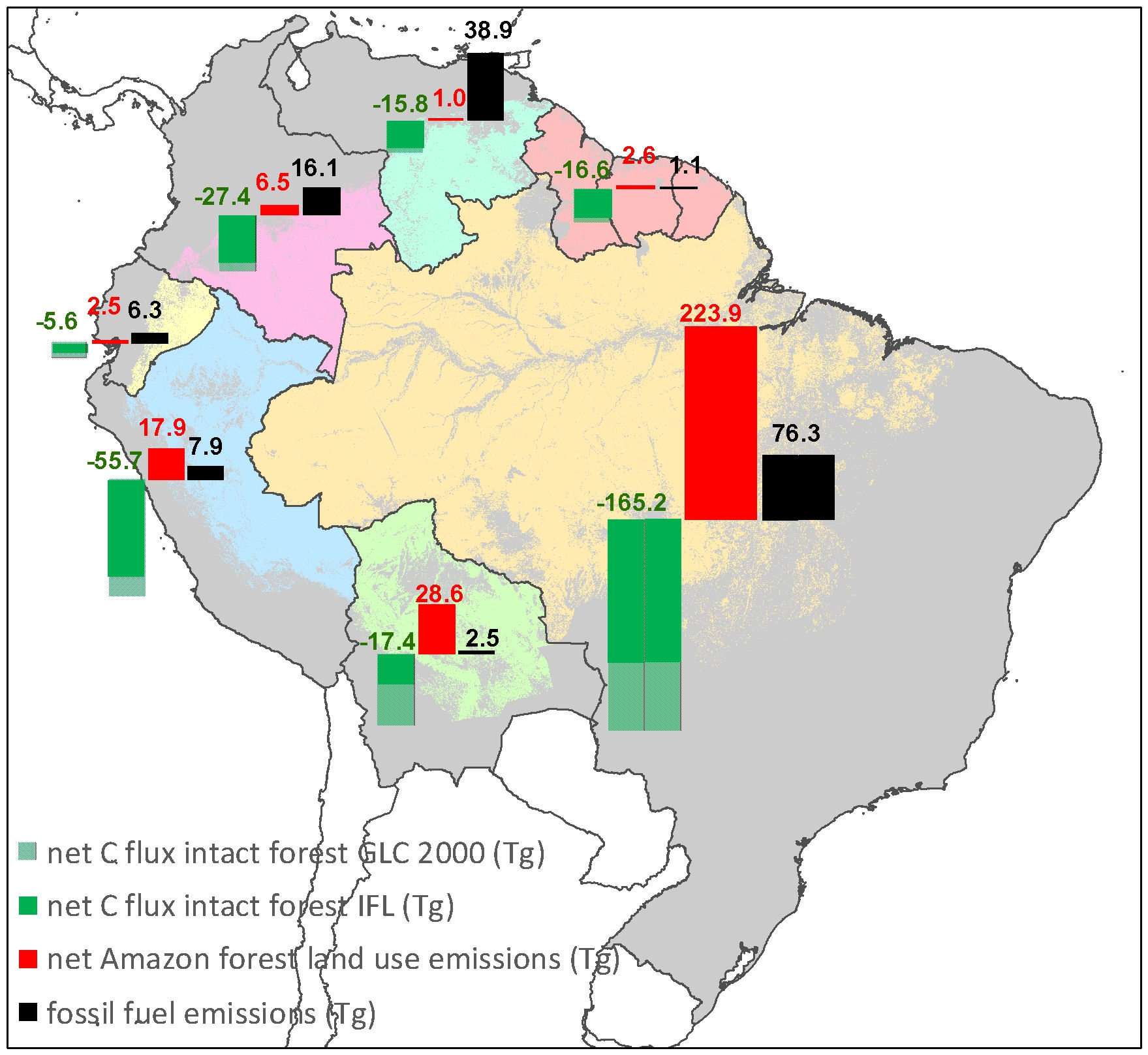

Supplement: Supplementary file 1 — Additional file 1. Detailed materials and methods; supplementary tables and figures. [file 13021_2016_69_MOESM1_ESM.docx]
